# Periostin: biology and function in cancer

**DOI:** 10.1186/s12935-022-02714-8

**Published:** 2022-10-12

**Authors:** Shima Dorafshan, Mahdieh Razmi, Sadegh Safaei, Erica Gentilin, Zahra Madjd, Roya Ghods

**Affiliations:** 1 Department of Molecular Medicine, Faculty of Advanced Technologies in Medicine, Iran University of Medical Sciences (IUMS), Tehran, Iran; 2 Oncopathology Research Center, Iran University of Medical Sciences (IUMS), Tehran, Iran; 3 Bioacoustics Research Laboratory, Department of Neurosciences, University of Padua, via G. Orus, 2b, 35129 Padua, Italy

**Keywords:** Periostin, POSTN, Cancer, Metastasis, Biomarker

## Abstract

Periostin (POSTN), a member of the matricellular protein family, is a secreted adhesion-related protein produced in the periosteum and periodontal ligaments. Matricellular proteins are a nonstructural family of extracellular matrix (ECM) proteins that regulate a wide range of biological processes in both normal and pathological conditions. Recent studies have demonstrated the key roles of these ECM proteins in the tumor microenvironment. Furthermore, periostin is an essential regulator of bone and tooth formation and maintenance, as well as cardiac development. Also, periostin interacts with multiple cell-surface receptors, especially integrins, and triggers signals that promote tumor growth. According to recent studies, these signals are implicated in cancer cell survival, epithelial-mesenchymal transition (EMT), invasion, and metastasis. In this review, we will summarize the most current data regarding periostin, its structure and isoforms, expressions, functions, and regulation in normal and cancerous tissues. Emphasis is placed on its association with cancer progression, and also future potential for periostin-targeted therapeutic approaches will be explored.

## Background

Periostin (POSTN) belongs to matricellular proteins, and since its first discovery in 1993, has become the subject of many studies in scientific research [1]. Matricellular proteins are a class of non-structural ECM proteins that are secreted into the extracellular environment and are expressed at low levels in most adult tissues. These proteins interact with cell-surface receptors and mediate cell and extracellular communications. Physiologically, periostin, a matricellular protein, regulates embryonic formation, tissue repair, ECM structure, formation and maintenance of bone and teeth [2], as well as other collagen-rich connective tissues subjected to mechanical stress, such as heart valves [3] and tendons [4]. In contrast, abnormal up-regulation of periostin expression has been observed in multiple pathological processes of various diseases, such as inflammatory diseases, fibrosis and tumor progression [5–9]. According to recent studies, periostin expression is significantly higher in cardiac disease and tumor tissues in the majority of cancers compared to normal tissues [10]. Periostin is overexpressed in a variety of solid epithelial tumors, and its interaction with cell-surface receptor integrins, which modulates intracellular signaling pathways, has a direct effect on cancer hallmarks [11]. It is up-regulated in metastasis and can influence the size and number of metastatic lesions, indicating that periostin plays a critical role in the formation and remodeling of cancer tissue microenvironments [11]. The main aim of this review is to explain the current knowledge about the role of periostin in tumor development and metastasis. First, we will briefly review its molecular properties and functions in a physiological state. Then, we will summarize the pathologic roles of periostin in tumorigenesis and metastasis, as well as recent insights into the functions of periostin in tumor microenvironments. Finally, we will discuss approaches that target periostin or related signaling pathways to develop novel cancer diagnostic and therapeutic strategies.

## Molecular structure of periostin

### Gene

#### Genomic organization

The periostin protein is encoded by the osteoblast-specific factor-2 gene, which is the official name for periostin. In humans, this gene is also known as POSTN, PN, OSF-2, and PDLPOSTN (Gene ID: 10631), and in mice, also known as Postn, OSF-2, Osf2, PLF, PN, A630052E07Rik, and AI747096 (Gene ID: 50706) [1]. The full length of human and mouse periostin genes encodes polypeptides of 836 and 838 amino acids, respectively. The mouse periostin gene is located on chromosome 3C with 25 exons (https://www.ncbi.nlm.nih.gov/gene/50706), while the human gene is located on chromosome 13's long arm (13q13.3) with 24 exons (https://www.ncbi.nlm.nih.gov/gene/10631) [12, 13]. Both terminal exons in the mouse and human are protein-coding regions [10]. The length of mouse periostin complementary DNA (cDNA) is 3187 bp, with an 18-bp 5′ untranslated region, a 733-bp 3′ untranslated region, and a 2436-bp open reading frame (ORF). The periostin gene in human spans approximately 36 kb [1].

#### Alternative splicing

**Fig. 1:**
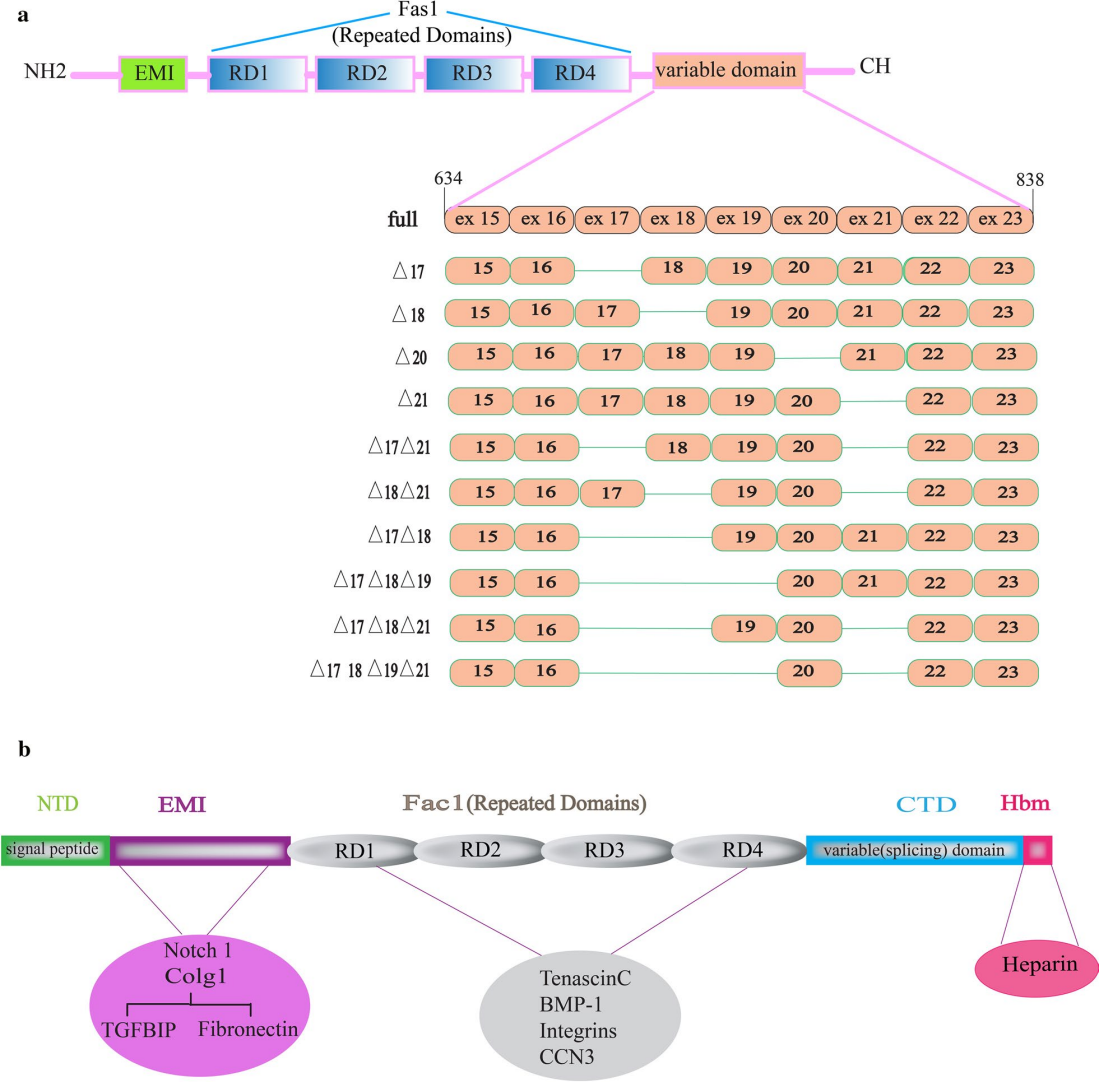
Periostin gene and protein structures. **a** Periostin splice variants' structures. In addition to full length, ten other isoforms have been investigated. **b** Protein domains of periostin and their interactions, The Signal Sequence, the EMI domain, the four FAS-1 domains, and the variable domain and a heparin-binding site (Hbs) that is located at the C-terminal end of the carboxyl-terminal domain (CTD) are all depicted

Except for the signal sequence and two regions in the C-terminal domain, mouse and human periostin have highly conserved amino acid sequence. Alternative splicing has resulted in insertions and deletions in C-terminal domain [1]. Totally, 10 splice variants as well as the full-length periostin have been discovered in human [14]. Mice originally displayed three isoforms, each resulting from the loss of one of three exons (17, 20, and 21) [15]. Then, the other isoforms were discovered by Morra et al. in human cancerous tissues, such as renal cell carcinoma and non-small cell lung cancer (NSCLC) [16, 17]. These splice variants include deletion of 1 exon (18), 2 exons (17 and 18 or 17 and 21 or 18 and 21), 3 exons (17, 18, and 19 or 17, 18, and 21), and 4 exons (17, 18, 19, and 21) (Fig. 1a). Furthermore, a periostin isoform known as periostin-like-factor (PLF) has been found in mice and humans. Periostin and PLF are nearly identical since they derive from the same gene and spliced mRNAs. The major differences between PLF and periostin reside in the C-terminal region [18]. PLF and Periostin differ in two distinct regions: between 673 and 699 aa and 785 and 812 aa. PLF has a sequence of 27 aa (673–699 aa, comprising exon 17) that is lacking in periostin. Also, there is a 28 aa (785–812 aa, comprising exon 21) sequence in Periostin that is not found in PLF [18]. The expression of these periostin variants significantly differs between tissues, for example, deletion of 1 exon (18) and deletion of 1 exon (17,21) [19] is the most prevalent isoform in renal tissue (Table 1) [18]. Full-length periostin is scarcely secreted and its levels are increased within the cell for fibrillogenesis during scar formation [12]. In contrast, the splice variants have a high secretion potential and can bind to specific integrin receptors, activating the Akt/PKB pathway via phosphorylation of focal adhesion kinase (FAK) and Phosphoinositide 3-kinases (PI3K) signaling pathways. This pathway is downstream of integrin signaling, which promotes cell migration and proliferation [20].

**Table1:** Periostin splice variants, including full-length one, and the tissues mainly expressing them

No.	Variants	Tissues	Refs.
1	Full-length	Fetal lung tissues, fetal kidney, normal bladder	[16, 17, 21]
2	∆18	Renal tissues	[16]
3	∆21	Fetal lung tissues, fetal kidney	[16, 17]
4	∆18∆21	Fetal lung tissues	[16]
5	∆17∆18	Fetal lung tissues, fetal kidney, NSCLC	[16, 17]
6	∆17∆21	Fetal lung tissues, normal renal tissues, periodontal ligament, periosteum, heart tissues after a myocardial infarction, NSCLC, renal cell carcinoma (RCC), bladder cancer, normal bladder	[16, 17, 21, 22]
7	∆17∆18∆19	Fetal lung tissues, fetal kidney, NSCLC	[16, 17]
8	∆17∆18∆21	Fetal lung tissues, NSCLC, bladder cancer, normal bladder	[16, 21]
9	∆17∆18∆19∆21	Fetal lung tissues, NSCLC	[16]
10	PLF	Bone, heart, and vascular smooth muscle cells, in mesenchymal cells in the periosteum and also in osteoblasts lining trabecular bone	[18]

### Protein

This protein was originally identified in the cDNA library of the mouse osteoblast cell line MC3T3-E1. At first, the mouse periostin cDNA was used as a probe to screen human placental and osteosarcoma cDNA libraries [1]. In humans, the ORF of placental periostin encodes an 87 kDa protein with 779 amino acids, whereas the ORF of osteosarcoma periostin encodes a 93.3 kDa protein with 836 amino acids and 838 amino acids in mice [23]. Using western blot analysis, it was found that the molecular weight of periostin is between 86 and 93 kD [24]. Comparing the amino acid sequences of mouse and human periostin reveals a homology of 89.2% for the entire protein and 90.1% for the mature form. However, the C-terminal region of the mature periostin protein shows a slightly lower degree of conservation than other regions, with 85.5% identity [1].

#### Secondary structure and domains

The secondary structure of periostin includes helix, turn and beta strand. In combination with multi-angle light scattering analysis and biochemical assays, the crystal structures show that human periostin mainly exists in solutions as a dimer. These dimeric forms can regulate the interaction of periostin in ECM remodeling [25, 26]. A periostin protein consists of an N-terminal signal peptide, which is required for secretion; a cysteine-rich region known as the EMI domain; a tandem of four repeated and conserved fasciclin-like (FAS1) domains; and a variable hydrophilic carboxy-terminal domain (CTD) (Fig. 1b) [12, 27, 28]. The signal peptide sequence for mouse periostin is 24 amino acids and 22 amino acids for human periostin respectively, but the mature form of periostin is 814 amino acids with a molecular weight of about 90 kDa in both mice and humans [1]. The EMI domain, composed of 75 amino acids and encoded by exons 2 and 3, is thought to participate in protein–protein interactions or protein multimerization, perhaps responsible for periostin dimers observed in some studies [29]. The FAS1 domains are encoded by exons 3 to 14. FAS1 is a 150-amino acid-residue evolutionarily ancient adhesion domain found in extracellular proteins. It is common to all living species [14, 30] and is present in many secretory and membrane glycosyl phosphatidyl inositol (GPI)-anchored proteins, where it binds to different ligands [14, 30]. Periostin Fas1 domains are homologous to protein fasciclin1; therefore, periostin belongs to the fasciclin family, that mainly includes midline fasciclin (MFAS) and TGF-induced protein (TGFBIp) [31]. The hydrophilic CTD, containing amino acids 634 to 838 (exons 16–22), has an arginine-rich heparin-binding site [27]. The multidomain structure of periostin may provide the basis for its functioning as a scaffold in the ECM milieu. Periostin has been reported to contribute to ECM remodeling by homophilic interactions with itself or heterophilic interactions with collagen, fibronectin, and tenascin-C [25].

The EMI domain can bind heterophilically to type I collagen, fibronectin, and Notch1 [32], and FAS1 domains can bind to tenascin-C, integrins (v3, v5), cellular communication network factor 3 (CCN3), and bone morphogenetic protein-1 (BMP-1). FAS1 domains are responsible for homophilic interactions of periostin [25]. It seems that the role of the EMI domain in mediating homophilic interactions remains slightly argumentative [25] (Fig. 1b).

## Expression

### Physiological expression of periostin

Periostin is expressed in variable amounts in a variety of normal tissues. Immunohistochemical analysis of mouse and human tissues showed that periostin is physiologically expressed in collagen-rich regions of connective tissues such as the periosteum, periodontal ligament [13], cardiac valve  [33, 34] lung [35, 36] and tendons [4]. It is also expressed in the aorta, stomach, lower gastrointestinal tract, placenta, uterus, and breast [37]. Periostin expression is typically scarce in adult tissues and elevated in stem cell niches in the mammary gland, bone, skin and intestine [38] and in some tissues during the fetal development  [3, 20]. For example, during the embryonic development and body growth, its expression is increased in periosteum and periodontal ligament tissues. In addition, embryonic fibroblasts and pericardial cells express high levels of periostin during cardiac development [15, 39]. Periostin expression was also found to be low in peripheral blood lymphocytes (PBLs), spleen, salivary gland, and thymus, while it was found to be high in skin and breast using qRT-PCR [37]. In some normal organs, such as the pancreas, liver, lymph nodes, lung, and colon, the periostin level is more homogenous, unlike the level of expression in ovaries. The increased expression of periostin in fibroblast-rich tissues, such as skin or breast, compared to tissues with lower fibroblast number, such as PBLs, spleen, pancreas, or liver, suggests that the normal tissues fibroblast may influence the expression of periostin in tissues [37].

### Pathologic expression of periostin in tissues and cancer cells

As mentioned, periostin expression is modest and balanced in some embryonic and adult normal tissues, but it is dysregulated and elevated in several pathological conditions. Dysregulation of periostin expression arises in fibrosis [35, 36, 40–42], wound healing tissues [43–46], inflammatory diseases such as arthritis, atherosclerosis [22, 23, 28], infarcted myocardium [15, 39], tumorigenesis, and metastasis [11, 22, 23, 28]. Ishikawa et al. discovered enhanced periostin expression in vitreous and retinal pigment epithelial (RPE) cells from the fibrous membranes of proliferative vitreoretinopathy (PVR) patients [40]. Idiopathic pulmonary fibrosis (IPF) patients' lung fibroblasts expressed periostin 3.5 times more than normal lung fibroblasts [36].

Many studies have also reported elevated periostin expression in primary tumors and metastatic lesions. Using Immunohistochemistry (IHC), Wu et al. found that colorectal cancer and liver metastasis lesions had higher periostin expression than non-malignant tissues. In this study, 30% of patients had increased periostin expression in CRC tissues than in paracancerous tissues. Using Western blotting, they revealed that the expression of periostin in cancer stem cells (CD133^+^) was higher than in CD133^−^ tumor cells [47]. It was found that increased periostin expression had a significant correlation with breast cancer progression using quantitative real-time PCR and western blot analysis. To elaborate, periostin expression was elevated in a stepwise manner from normal tissue to ductal carcinoma in situ (DCIS) tissue and invasive breast cancer (IBC) tissues [48]. Around 60% of melanoma metastatic tumors in the liver or lymph nodes overexpress periostin, although periostin expression is not increased in primary tumors. Also, it was shown that the main sources of periostin are melanoma and stromal cells [37]. Sasaki et al. used chemiluminescence to evaluate periostin serum levels in thymoma patients (stages 1–4) and healthy controls. They found that only stage IV patients had significantly higher periostin serum levels than controls [49]. Also, with chemiluminescence and in situ RNA hybridization, they detected higher serum levels of periostin protein and the periostin gene in stromal tumor tissues of breast cancer patients, and a correlation was seen between increased serum levels of periostin and bone metastasis [50]. Although periostin overexpression has been seen in a variety of human malignancies, it is not a common characteristic of tumors. For instance, Tilman et al. used qRT-PCR to show that periostin is not expressed in hematological malignancies such as leukemia and myeloma, whereas there is a significant increase in periostin expression in pancreatic, liver, and NSCLC tumors [37].

Periostin expression is reported to be negative or low in most cancer cell lines [8, 51–54], and only a small number of cell lines show high or moderate expression (Table 2). Although Tai et al. reported that periostin expression significantly increased in colorectal cancer tissue and metastatic liver lesions, they observed unexpected results (negative expression) in RNA expression in a variety of cell lines, including colon cancer (HCT 116, RKO, SW-620, and HT 29), breast cancer (MDA 435, MCF-7), uterine sarcoma (MES-SA, MES-SA/DX5), pancreatic cancer (MIA PaCa-2), except in mesothelioma (JMN1B) and normal colon cell line (CCD-112CoN), that expression was increased [55]. Another study reported a moderate expression level in A172 glioblastoma (45 ± 4) and a significant expression level in Hs578T breast cancer (3693 ± 86) and LB831 bladder carcinoma (1748 ± 74) using quantitative RT-PCR [37]. In addition, increased periostin expression was detected in the bladder cancer cell lines MIBC, J82, TCC-SUP, and UMUC3 [56]. Table 2 shows the periostin expression in various cancer cell lines, and Table 3 shows the periostin expression in various cancer tissues.

**Table 2:** Periostin expression in different cancer cell lines

Origin of cancer	Cell line	Periostin expression	Detection assay	Refs.
Pancreatic	Panc-1	Neg	qRT-PCR	[52] [53] [8] [37]
	Capan-1	Neg		
	BxPC-3	Neg	RT-PCR	
	MiaPaCa-2	Neg		
	AsPC-1,	Neg		
	Capan-2	Neg		
	AsPC1	Neg		
	SW1990	Neg		
	Panc-1	Neg		
	HPAF	Neg		
	CFPAC-1	Neg		
	Colo-357	Neg		
	SU86.86	Very weak		
Colorectal	HT29	Neg	RT-PCR	[57] [37] [51]
	SW620	Neg		
	LS174T	Neg		
	SW480	Neg		
	LB108	Neg	qRT-PCR	
	SW837	Neg	Northern blot RT-PCR	
Bladder	SBT31A	Neg	RT-PCR Northern blot	[58] [56] [37]
	HT1197	Neg		
	T24	Neg		
	J82	High	qPCR	
	UM-UC-3	High		
	LB108	High		
	LB831	High		
	TCC-SUP	High	qPCR Western blot	
	UMUC3	High		
	MIBC	High		
Small cell lung cancer (SCLC)	H69	Intermediate	Northern blot RT-PCR	[51] [37]
	LB85	Weak	qRT-PCR	
	LB92	Weak		
Non-small cell lung cancer (NSCLC)	LB37-1	Weak	qRT-PCR	[51] [37]
	RERF-LC–MS	Weak	Northern blot RT-PCR	
	VMRC-LCD	Neg		
	CADO-LC4	Neg		
	CADO-LC29	Neg		
	A549	Neg		
	CADO-LC46	Neg		
cervical cancer cell	HeLa	Weak	qRT-PCR	[51] [37]
	C33A	Weak	Northern blot RT-PCR	
	SiHa	Neg		
	CaSki	Neg		
Osteosarcoma	SaOS-2	Weak	RT-PCR	[51] [37]
	U2OS	Weak	qRT-PCR	
Breast cancer	MCF7	Neg	qRT-PCR	[51] [37] [59]
	MCF10A (MI)	Neg	qRT-PCR Western blot	
	MCF10AT1k.cl2(MII)	Neg		
	MCF10CA1h(MIII)	Weak		
	Hs578T	High	qRT-PCR	
	BT549	High	qRT-PCR	
	SUM1315	High	qRT-PCR	
	SUM159	High	qRT-PCR	
Ovarian carcinoma	A2780	Weak	western blot ELISA	[51] [37]
	OVCAR-3	Neg		
	OV2008	Neg		
	PA-1	Weak	qRT-PCR	
Renal cell carcinoma	LB1047	Weak	qRT-PCR	[37]
	BB64	Weak		
Melanoma	MZ2	Weak	qRT-PCR	[37] [51]
	LB39	Weak		
	LB2586-7	Weak		
	LB2201-3	Weak		
	A375	Weak		
	MeWo	Neg	Northern blot RT-PCR	
Endometrial	S3	Neg	Northern blot RT-PCR	[51]
Wilms’ tumor	G401	Weak	Northern blot RT-PCR	[51]
Bladder cancer	T24	Neg	Northern blot RT-PCR	[51]
Fibrosarcoma	HT1080	Neg	Northern blot RT-PCR	[51]
Neuroblastoma	NB-1	Neg	Northern blot RT-PCR	[51]
Gastric cancer	AZ521	weak	Northern blot RT-PCR	[51]
Pancreatic cancer	MIA-PaCa-2	Neg	Northern blot RT-PCR	[51]
Hepatocarcinoma	Huh-7	Weak	qRT-PCR	[37]
Stomach cancer	MZGC3	Neg	qRT-PCR	[37]
Neuroblastoma	NB-1	Neg	Northern blot RT-PCR	[51]
Ewing sarcoma	LB96	Neg	qRT-PCR	[37]
Glioblastoma	A172	Intermediate	Northern blot qRT-PCR	[51] [37]
Fibrosarcoma	HT1080	Neg	Northern blot RT-PCR	[51]
Rhabdomyosarcoma	LB23-1	Weak	qRT-PCR	[37]

**Table 3:** Periostin localization in various cancer tissues

Cancer type	Localization	Main findings	Refs.
Breast	Tumor stromal cells, CAFs	Expression of PN in CAFs was significantly increased in a stepwise manner from FC to DCIS and IDC	[60]
	Tumor epithelial cells, tumor stromal cells	Expression of PN protein and mRNA is higher in cancer tissues than in adjacent normal tissues Both epithelial and stromal PN expression were significantly increased in a stepwise manner from normal breast tissue to DCIS and IBC Distant metastatic relapse-positive patients had higher epithelial PN expression than distant metastatic relapse-negative patients	[48]
Head and neck	Tumor stromal cells, especially CAFs and adjacent normal tissues	Expression of PN in the stroma of cancer tissues was much higher than adjacent normal tissues	[61]
Osteosarcoma	Tumor cells	Expression of PN in Cytoplasm of tumor cells	[62]
Glioma	Tumor cells	Expression of PN in either the nucleus or cytoplasm	[63]
Ovary	Tumor epithelial cells		[64]
	Tumor epithelial cells, tumor stromal cells		[65]
	Tumor stromal cells		[66]
Oral squamous cell carcinoma	Tumor epithelial cells		[67]
Cutaneous squamous cell carcinomas	Stroma	PN levels was significantly increased in a stepwise manner from SCCIS, LR-cSCC, HR-cSCC to RDEB SCC	[68]
Nasopharyngeal carcinoma	Stroma		[67]
Colorectal	Tumor cells		[69]
	Stroma (in metastatic tumors in addition to primary tumors) and low expression in cancer cells		[57]
	Stroma		[70]
	Tumor cells	Expression of PN in cytoplasm and membrane of the CRCs and metastasis tumors	[47]
Non-Small Cell Lung Carcinoma (NSCLC)	Tumor epithelial cells, tumor stromal cells	Expression of stromal PN was consistently enhanced in the pseudo-basement membrane surrounding carcinoma cells	[71]
	Mesenchymal tissue surrounding the tumor cells	Expression of PN in were elevated in the mesenchymal areas but not in the cancer cells themselves	[72]
	Areas that co‑localized with myofibroblasts		[73]
Pancreatic	Tumor epithelial cells, tumor stromal cells	PN staining in the neoplastic stroma was seen in up to 80% of tumors, while PN staining in the neoplastic epithelium was found in only 30% of tumors	[54]
	Tumor stromal cells	Expression of PN was found in stromal cells adjacent to the pancreatic epithelial cells	[52]
	Stromal cells (in metastatic tumors in addition to primary tumors)		[8]
Prostate	Tumor stromal cells, tumor epithelial cells (some cases)		[74]
	Tumor stromal cells (in metastatic tumors in addition to primary tumors), tumor epithelial cells	Expression of PN in cytoplasm of tumor epithelia	[75]
	Tumor epithelial cells, tumor stromal cells	Expression of PN was higher in epithelium (well-differentiated tumor), stroma (poorly differentiated tumors) and bone metastases	[76]
	Tumor epithelial cells, tumor stromal cells	Expression of PN in stroma was found to be significantly greater than in epithelial cells	[77]
Liver	Tumor epithelial cells, tumor stromal cells	Expression of PN in HCC: Epithelial: 19 ⁄ 91 (20.9%) and strong stromal: 10 ⁄ 91 cases (11%) In BDC: Epithelial: 39 ⁄ 116 (33.6%) BDC and stromal: 78 ⁄ 116 (67.2%)	[78]
	Tumor cells	Expression of PN was mainly in the cytoplasmic area of HCC cells	[79]
Bladder	Epithelial	Expression of PN in Cytoplasm	[56]

CAFs: Cancer-associated fibroblasts; IDC: invasive ductal carcinoma; DCIS: non-invasive ductal carcinoma in situ; FC: fibrocystic change; IBC: invasive breast carcinoma; HCC: hepatocellular carcinoma; BDC: bile duct carcinomas; CRC: colorectal cancer; cSCC: cutaneous squamous cell carcinoma; SCCIS: cSCC in situ; LR-cSCC: low-risk cSCC; HR-cSCC: high-risk cSCC; RDEB cSCC: cSCC in recessive dystrophic epidermolysis bullosa patients

## Regulation of expression

### Physiologic and pathologic regulation of periostin expression

Various factors regulate periostin expression in physiologic or pathologic conditions [28]. Transcription factors, which play a role in the differentiation of pluripotent mesenchymal cells into osteoblastic lineage, are one of the factors that influence periostin expression [11]. Transcription factors TWIST1 and 2 are important regulators of periostin expression in the physiological state. Franco et al. were the first to identify an association between periostin and twist in a very rare autosomal recessive syndrome, Setleis syndrome (OMIM 227260), a disorder characterized by abnormal facial growth. They discovered that mutations in the TWIST2 led to the reduction of periostin expression in the fibroblasts of these patients [80]. In this regard, Oshima et al. reported a "Twist box" response element in the periostin promoter and showed that the Twist can bind the Twist-box sequence on the periostin promoter and upregulate periostin expression [81]. They also used Northern blot, RT-PCR, and gene expression profiles to show the co-expression of periostin and Twist in bones, and the role of Twist in periostin up-regulation during osteogenesis [81]. Using the Cancer Genome Atlas database, Hu et al., found a correlation between the expression of periostin and Twist/Snail in the lung cancer tissues [82]. Interestingly, the expression of periostin was suppressed using *Twist* shRNA in prostate cancer cell lines [83]. c-Fos/c-Jun (AP-1) is another transcription factor that regulates periostin expression. IHC and in situ hybridization were used to reveal a correlation between the expression of c-Fos/c-Jun and periostin in the fibrous component of human fibrous dysplasia lesions [84]. Also, periostin expression in the sclerotic lesions formed in transgenic mice overexpressing c-fos is similar to that found in fibrous dysplasia, in all lesion, transformed osteoblast expressed elevated periostin level, in contrast to normal osteoblasts. Consequently, a direct correlation between increased periostin and c-fos expression was discovered [84]. ChIP tests indicate that tumor protein 73 (P73) is another transcriptional factor that binds to the promoter of periostin in glioblastoma cells and regulates glioblastoma cell invasion via controlling periostin synthesis. In addition, bioinformatics analysis revealed that the p73/periostin axis is predictive of a poor prognosis in various cancer types [85]. Slug and Sox9 are two additional transcription factors involved in the regulation of periostin expression, and their overexpression induce tenascin-C and periostin expression [11]. Besides transcription factors, hormones play an important role in periostin expression. Estrogen and parathyroid hormones modulate periostin expression [86–88]. Also, angiotensin II induces the expression of periostin in rat cardiac fibroblasts [89] and vascular smooth muscle cells [90]. Other effective regulators are growth factors; TGF-β increase periostin expression in osteoblasts [13], human periodontal ligament cells [91, 92], gingival fibroblasts [93], and kidney mesangial cells [94]. BMP-2 is an effective growth factor that has been shown to regulate periostin expression in endocardial cushion mesenchyme [34, 95]. After BMP2 activation, Smad1/5/8 and Twist-1 are induced, which eventually leads to overexpression of periostin [81, 96, 97]. Various studies have shown that cytokines, such as IL-4, IL-13, TGF-1/3, PDGF-, bFGF, and TNFα, promote periostin expression, making cytokines an important regulator of periostin expression [12, 34, 95]. Finally, FAK, PI3K, Akt, ERK, NF-kB, and STAT-3 are the downstream signaling pathways that modulate periostin expression (Table 4) [12].

**Table 4:** Molecules and signaling pathways influencing periostin expression

Transcription factors	Twist-1 Twist-2	Snail	c-Fos/c-Jun	p73	Sox9	Slug	[11, 81, 82, 84, 85]
Growth factors	IL 3, 4, 6, and 13	TGFβ _1/3	FGF 1	BMP-2	PDGF	-	[12, 34] [34, 95]
Hormones	Parathyroid	Estrogens	Angiotensin II	-	-	-	[86, 87, 88]
Downstream signals	FAK	PI3K	Akt	ERK	NF-kB	STAT-3	[12]

## Localization of periostin in normal and cancerous tissues

Periostin is an ECM protein that is mostly secreted in the periosteum (a thick layer of vascular connective tissue encircling the bone surfaces) and periodontal ligament (a group of specialized connective tissue fibers). It is often expressed by fibroblasts in normal tissues and secreted into the surrounding ECM and sometimes remains in the cytoplasm and nucleus at the cellular level [38, 51, 98]. Indeed, periostin plays different roles depending on its localization. A study, for example, found a difference in periostin localization in healthy skin and remodeled dermis. In healthy skin, periostin is mainly localized in the epidermis and in the nuclei of keratinocytes. However, in remodeled dermis, periostin was found mostly in the ECM and near large fibrils rounding off the cells [99]. Also the localization of various isoforms of periostin are different. For instance, unlike the intact form of periostin, a highly conserved isoform in mice, humans, and zebrafish is easily secreted outside the cell. Exons 17 and 21 are deleted in this isoform, which can be found in the periodontal ligament, periosteum, and heart tissues following a myocardial infarction [16, 22, 100].

In malignant tissues the localization of periostin is altered. In a study of 30 NSCLC samples, periostin expression was detected in the cytoplasm of tumor epithelia (periostin tumor) and in the cytoplasm of fibroblasts or extracellular matrix (ECM) (periostin stroma) [16]. In many cancers, periostin is mostly found in the cancer stroma, but it is also detected in epithelial cancer cells and in both epithelial and stromal cancer cells [8, 52, 57, 60, 67]. Using IHC, the majority of periostin was detected in the stroma of nasopharyngeal carcinoma (NPC) [67], the DCIS and the IBC. In breast cancer, distant metastatic relapse-positive cases had a higher frequency of epithelial expression than distant metastatic relapse-negative patients [48]. Qin et al. used western blot analysis, real-time PCR, semi-quantitative RT-PCR analyses, and IHC in order to compare the levels of periostin in normal oral epithelial cells, cancer-associated fibroblasts (CAFs) and normal fibroblasts (NFs) isolated from head and neck cancer (HNC) tissues, as well as cell lysates from 6 HNC cell lines [61]. They found that the expression of periostin was somewhat increased in HNC cells compared to normal oral epithelial cells. Periostin expression was significantly higher in CAFs than in NFs. Utilizing confocal microscope imaging also confirmed this data [61]. As a secretory protein, periostin was found in CAF culture media using ELISA. Overall, the main source of periostin in tumor tissues is the cancer stroma, especially CAFs [8, 52, 57, 60, 61, 67]. Table 3 summarizes periostin localization in various cancer tissues.

## Function

### Role of periostin in normal tissues

As described above, periostin is physiologically expressed in the periosteum and periodontal ligament, and it has a function in bone and tooth formation and maintenance of structure [13, 101, 102]. In the non-embryonic period, periostin covers the outer surface of bones and is responsible for the growth of bone diameter, bone strength, and cortical thickness [103, 104]. During embryonic development and body growth, the activity of the periosteum increases. Also, periostin is associated with mesenchymal differentiation in the embryonic heart development [10, 105, 106]. Periostin's features make it a key player in the regulation of cell behavior and ECM organization. It has been demonstrated that periostin enhances the ECM intermolecular interactions, leading to increase the mechanical strength of connective tissues [12].

At the molecular level, FAS1 domains interact with cells, while the N-terminal EMI domain and CTD interact with ECM proteins. The EMI domain can bind to type I collagen, fibronectin, and Notch1 [32], and the FAS1 domain binds to tenascin-C, BMP-1, CCN3 and integrins αvβ3 and αvβ5 [107, 108]. The binding of periostin to integrins αvβ3 and αvβ5 in osteoblasts and numerous types of normal and malignant cells activates the FAK, PI3-Kinase, and AKT signaling pathways, resulting in cell migration [69, 109, 110]. These findings indicate that periostin can serve as a prosurvival protein in many cellular conditions. Because CCN3 and Notch 1 are involved in stemness maintenance, periostin binding to them represents another aspect of periostin's function [100].

The "periostin switch" is a new periostin activity. Interestingly, periostin in early step of expression acts as a positive regulator of collagen synthesis while the function of periostin alters to collagen cross- linking after cutting of its C-terminal domain [12]. Moreover, the EMI domain of periostin is required for its multimerization, which helps collagen cross-linking through forming a meshwork structure with fibronectin and tenascin-C [107]. Briefly, periostin serves as a scaffold for BMP-1 and collagen in order to promote collagen cross-linking. BMP-1 cleaves the inactive form of LOX to generate the active form, which catalyzes the covalent cross-linking of collagen molecules [111, 112]. Periostin-null mice exhibited abnormal collagen fibrillogenesis in the periosteum and a decrease in collagen cross-linking in the skin, tendons, and heart [11]. Collagen formation and collagen cross-linking mediated by periostin is a natural process required for mechanical strength of collagen-rich connective tissues. Due to similarities between periostin and the fasciclin I family, periostin involves in both cell adhesion and signal transduction like other fasciclin I family members [13]. In summary, periostin is a key regulator of ECM and tissue remodeling and affects cell migration and adhesion and EMT [28].

### Role of periostin in cancer development and progression

**Fig. 2:**
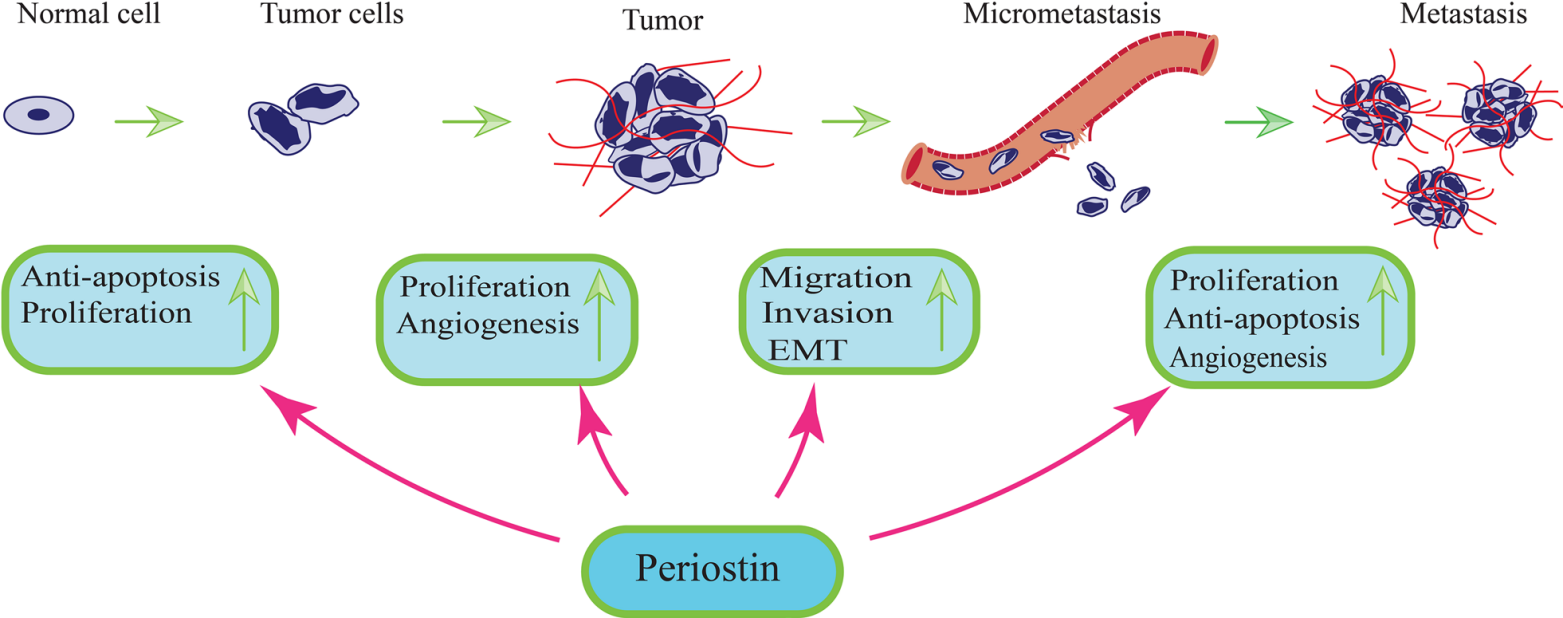
Possible roles of periostin in tumorigenesis. Periostin could be involved in the transformation of normal cells into metastatic tumors by preventing apoptosis, and promoting cell proliferation, angiogenesis, migration, EMT, and invasion

**Fig. 3:**
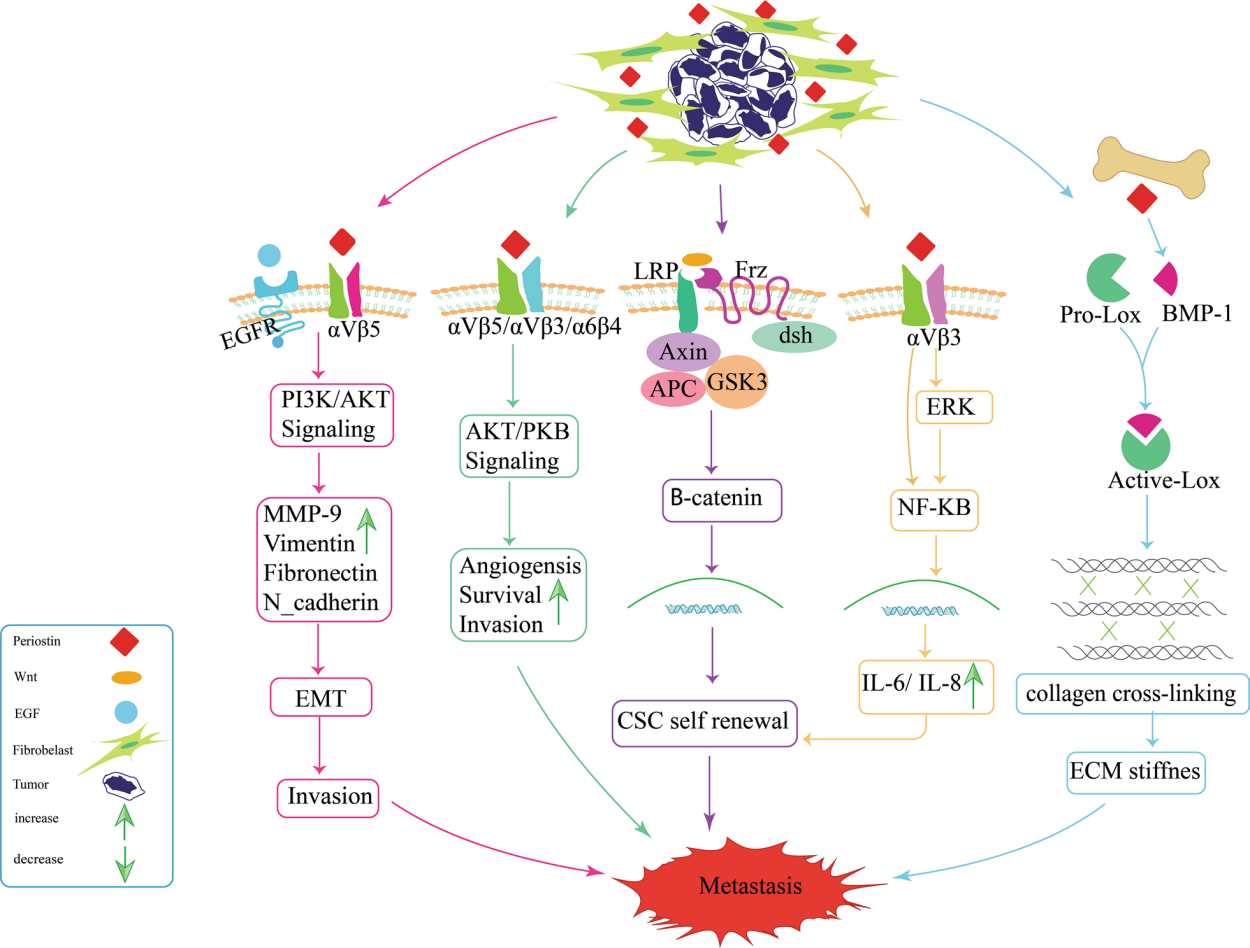
Overview of the signaling pathways involved in periostin-induced metastasis. **a** Periostin via cross-talk between integrin and EGFR activates the PI3K/Akt signaling pathways and increases MMP-9, vimentin, fibronectin, and N-cadherin. So it affects EMT and increases invasion and metastasis. **b** Periostin promotes angiogenesis, cell survival, invasion, and metastasis by activating the Akt/PKB signaling pathways. **c** Periostin activates the *ß*-catenin dependent pathway by inducing wnt binding to its receptors, resulting in csc self-renewal and metastasis. **d** The binding of periostin to its receptor activates the NF-ƙB pathway, inducing IL-6 and IL-8 transcription and, ultimately, csc self-renewal and metastasis. **e** As a result of periostin's interaction with BMP-1, which increased the proteolytic activity of LOX, collagen cross-linking and ECM stiffness are enhanced, resulting in metastasis

Periostin has functions in cancer, fibrosis, and inflammatory diseases such as infarcted myocardium, arthritis, atherosclerosis, and asthma. The dysregulation of periostin expression in several cancers indicates that it plays an important role in cancer development and progression (Fig. 2). It binds to integrins on cancer cells, activating the Akt/PKB and FAK signaling pathways (Fig. 3). As a result, angiogenesis, invasion, metastasis and cell survival increase [113]. As mentioned above, periostin transcript is subjected to alternative splicing. It has been shown that the different isoforms of periostin are expressed in various cancer, including pancreatic, colon, breast, lung, and renal cell carcinoma (RCC) [10, 17, 69]. Some lines of evidence indicate that these isoforms are associated with cancer progression [21]. Keda-Iwabu et al. showed that exon 17 is required for breast cancer growth and metastasis via binding to wnt3a [114]. The role of periostin in cancer will be discuss in following sections.

#### Tumor growth

It is well known that uncontrolled tumor growth results in an imbalance between cell proliferation and cell death [115]. Cancer cells can disrupt the balance between pro- and anti-apoptotic factors to increase cell survival in the presence of environmental signals [69]. Given the well-known periostin functions in cancer, the question remains as to which mechanism is at work. In fact, periostin induces tumor growth via promoting cell proliferation or escaping apoptosis. To elucidate the role of periostin in the progression of cancer cell proliferation, Hong et al. demonstrated that periostin has a significant effect on increasing cell proliferation [116]. Also, Kikuchi et al. showed that periostin enhances the proliferation of OCUM-2MLN and OCUM-12 diffuse-type gastric cancer cell lines via ERK phosphorylation of the MAP kinase pathway [117]. Tai et al. revealed that treatment of MIP101 colorectal cancer cells with periostin results in a significant increase in cell proliferation [55]. Contrary to previous findings, Shao et al. reported that periostin overexpression is not associated with an elevated proliferation rate. To create stable cell lines that overexpress periostin, they utilized three tumor cell lines that lack detectable levels of endogenous periostin: the human kidney epithelial cell 293T, the highly metastatic mouse melanoma cell B16F1, and the metastatic human breast cancer cell MDA-MB-231. Surprisingly, proliferation rate of these periostin-producing cells were slower than control cells in vitro [118]. In addition, when periostin-producing cells were transplanted as xenografts into immunocompromised SCID-Beige mice, they showed a phenotype of tumor growth and angiogenesis. Ultimately, they showed that periostin promotes angiogenesis by elevating VEGF receptor 2 expression in endothelial cells through an integrin αvβ3 -FAK-mediated signaling pathway [118]. The findings of a research conducted by Kudo et al. on Human Oral Squamous Carcinoma cell line2 and Human Oral Squamous Carcinoma cell line3 (HSC2, HSC3) also showed that periostin overexpression does not enhance cell proliferation, but it significantly enhances tumor cells invasiveness [9]. In general, it should be noted that the effect of periostin on the proliferation of cancer cells varies depending on the cell types. Some studies have found that inducing periostin expression in tumor cells can prevent apoptosis and increase survival under several stress conditions, as observed in pancreatic cancer cells [119–121]. Periostin can affect cancer cells to survive under hypoxic conditions by inhibiting stress-induced apoptosis [23, 122]. It has been also showed that periostin reduced apoptosis and increased chemoresistance in human colorectal cancer cells by upregulating the anti-apoptotic protein survivin and activating the PI3K/Akt/survivin pathway [123]. In pancreatic ductal adenocarcinoma (PDCA), periostin is associated with gemcitabine-chemoresistance [124]. Periostin expression upregulated in triple negative breast cancer xenograft following chemotherapy in tumor cells and promoted invasion of residual of tumour cells. Suppression of periostin inhibited the growth and invasion of mesenchymal tumor cells [19].The presented data show the role of periostin in cancer cell proliferation, survival and tumor growth [23].

#### Angiogenesis

Several molecules activate angiogenic signaling pathways in human microvascular endothelial cells [125]. Periostin has been identified as a new and powerful angiogenic factor for tumor growth [118, 126]. Colorectal cancer is an example in which periostin significantly increases metastatic growth by promoting human endothelial cell survival and inducing angiogenesis. Indeed, activation of the Akt/PKB cell survival pathway through periostin binding to αvβ3 integrins protects tumor and endothelial cells from stress-induced death and enhances angiogenesis [69]. In human breast cancers, periostin overexpression also leads to a substantial increase in angiogenesis. Breast cancer cell lines overexpressing periostin enhance tumor angiogenesis in vivo by activating FAK signaling via integrin-v3 and upregulating the VEGF receptor Flk-1/ KDR in endothelial cells [118]. Periostin expression has been reported to correlate with vascular endothelial growth factor-C (VEGF-C) expression in both the tumor and serum of HNC patients [127]. In addition, periostin stimulated tube formation of endothelial cells independently of VEGF-C via the Src and Akt pathways, and a potential correlation was found between periostin and lymphatic status in periostin-overexpressing xenograft tumors and HNSCC patients [127]. Oral cancers and NSCLC are two examples of periostin–integrin interactions that promote angiogenesis in endothelial cells [7, 128].

#### Invasion and metastasis

Tumor invasion and metastasis are multifaceted, uncontrolled, and complex processes in tumor development [69], and cancer cells utilize a variety of strategies which lead to the establishment of secondary tumor sites. These multistep process require interactions between cancer cells, stromal cells, and ECM. Changes in ECM components within the tumor microenvironment have a considerable impact on the metastatic process [129]. The question now is, how periostin promote metastasis? Periostin could be involved in metastasis through ECM remodeling, premetastatic niches [69, 130], cancer stem cell niches [38, 131], and perivascular niches formation [132] and also fibrotic microenvironment establishment [133]. In addition, periostin regulates critical metastatic processes, such as EMT, motility, tumor cell survival, angiogenesis, and tumor cell stemness [53]. In a study, the correlation between periostin and oral squamous cell carcinoma (OSCC) metastasis and invasion was evaluated by measuring the periostin mRNA level in tumor tissues. According to this finding, 68% of OSCC patients have elevated levels of periostin mRNA, and the majority of periostin-positive OSCC primary tumors have metastasis [7]. Periostin has also been shown to play an important role in establishing the number and size of liver metastasis in mice with colon cancer [69]. In addition, increased expression of periostin promotes tumor metastasis in gastric, breast, and colon cancers [47, 50, 57, 134, 135].

EMT is a critical step in tumor metastasis, and periostin is involved in both EMT and metastasis [136, 137]. Hong et al. used RT-PCR to evaluate the mRNA levels of EMT markers in periostin transfected A549 cells and revealed that periostin increases vimentin and N-cadherin expression while decreasing E-cadherin expression. As a result, they hypothesized that periostin promotes migration via activating the EMT pathway [116]. Periostin-sustained expression in 293T cells (tumorigenic but non-metastatic) resulted in fibroblast-like transformation with increased expression of vimentin, epidermal growth factor receptor (EGFR), and matrix metalloproteinase-9 (MMP-9) in animal models. Also, periostin-engineered 293T cells promoted the development of metastases in immunodeficient mice [138]. Also, ectopic periostin-expressing cells increased cell migration, invasion, and adhesion by 2–ninefold through cross-talk between the integrin and EGFR signaling pathways (Fig. 3) [138]. It was shown that periostin enhances angiogenesis and metastasis via activating the Akt/PKB pathway, which protects tumor and endothelial cells against stress-induced cell death [69] (Fig. 3). Periostin modulates EMT and promotes migration and metastasis via the AKT signaling pathway in pancreatic cancer cells in a dose-dependent manner [52]. Furthermore, it has been found that periostin is an EMT regulator and induces the expression of MMP-9, MMP10, and MMP-13, leading to ECM destruction, which is important for local tumor spread and metastasis [28, 127, 139, 140].

Periostin is also critical for the formation of pre-metastatic niches (PMN). According to previous studies, periostin may serve as a chemoattractant for cancer cells and is important for metastatic cell colonization by conditioning the premetastatic niche [141]. Interestingly, periostin could also be delivered to metastatic sites by tumor exosomes, thereby promoting metastasis by priming PMN of the target tissue before tumor cell entrance [141]. Vardaki et al. found that the protein composition of exosomes derived from metastatic human breast cancer cell lines was significantly different from that of exosomes derived from non-metastatic cell lines [141]. Abundance of adhesion proteins and periostin was found by analysis of exosome proteomic profile of metastatic cell lines [141]. It has been shown that in animal model, knockdown of periostin reduces metastasis burden suggesting that periostin is involved in premetastatic nichs formation [142]. In a mouse model of MMTV-PyMT, periostin is required for the formation of an immunosuppressive pre-metastatic niche in the lungs during breast cancer metastasis by recruiting MDSCs and activating ERK, AKT, and STAT3 [130]. Also, using the pyMT mouse model of breast cancer, it was shown that periostin as the stromal component of the metastatic niche plays an important role in metastasis progression, and a reduction in the number and size of pulmonary metastases was found in periostin ^−/−^ mice [38]. CCL2 plays a vital role in PMN formation through the recruitment of bone marrow-derived cells (BMDCs). In an interesting study, it was shown that periostin upregulates CCL2 expression in B cell acute lymphoblastic leukemia (B-ALL) cells via activating the integrin-ILK-NF-kB pathway. Also, it was found that leukemia cell-derived CCL2 activates STAT3 to increase periostin expression in bone marrow-derived cells (BMDCs). These results revealed a positive correlation between periostin and CCL2 levels in B-ALL patients, which contributes to the increased leukemia burden [143].

At the molecular level, periostin acts through Akt/PKB, Wnt, and FAK/Src signaling pathways to promote metastasis [53]. For example, it stimulates the Akt/PKB and FAK/Src signaling pathways via the αvβ3 integrins, promoting angiogenesis, invasiveness, cellular survival, and reducing apoptosis, eventually increasing metastatic potential (Fig. 3) [69]. Periostin also promotes metastasis by increasing Wnt signaling and promoting stem cell survival. Periostin is also implicated in bone metastases via a different pathway. Interaction of periostin with BMP-1 enhances the activity of LOX resulting in ECM stiffness. Therefore, periostin is most likely involved in bone metastases via the stimulation of BMP-1 and LOX activities [24, 112] (Fig. 3).

### Tumor suppressor

As mentioned above, overexpression of periostin is associated with enhanced invasiveness in most cancers but not in bladder cancer [58]. Even though some reports indicate that periostin is overexpressed in bladder cancer and is associated with a poor prognosis in muscle-invasive bladder cancer [56]. Using IHC, Kim et al. revealed that periostin expression is lower in bladder cancer tissues than in normal bladder tissues and lower expression was inversely correlated with tumor grade [58]. Periostin affects EMT and cell invasiveness differently in prostate and bladder cancer cells. In prostate cancer, periostin increase Akt phosphorylation, which leads to the upregulation of Snail, which is a negative regulator of E-cadherin causing prostate cancer cells to invade more. In contrast, in bladder cancer, periostin suppressed Akt phosphorylation, followed by downregulation of Twist, an E-cadherin negative regulator, leading to an increase in E-cadherin expression and a decrease in bladder cancer cell invasion [144]. In gastric cancer, epithelial-derived periostin acts as tumor suppressor by stabilizing p53 and E-cadherin proteins via the Rb/E2F1/p14ARF/Mdm2 signaling pathway [145], whereas periostin-derived stroma significantly enhances the proliferation of gastric cancer cells [117, 145]. Some studies demonstrate that periostin has a dose-dependent biphasic effect. At 150 ng/ml, periostin can prevent EMT and decrease in vitro cell migration in pancreatic cancer cells, leading to metastatic suppression in vivo. In contrast, at high levels of periostin (1 μg/ml), cell migration is stimulated by activation of Akt [52]. In addition, several studies on lung cancer show that periostin is unexpectedly downregulated in malignant tissues compared to normal tissues [51]. Based on these findings, it appears that periostin derived from different cell types may play distinct biological functions in the development of tumors. On the basis of these findings, it seems that periostin derived from different cancer cell types may bind to various integrin receptors. Moreover, cancerous tissues contain several spliced isoforms of periostin. These factors may explain the contradictory function of periostin as a tumor suppressor or progressor [1, 13, 145].

#### Stemness

According to recent research, there is a close interaction between cancer stem cells (CSCs) and metastatic niches [146]. Periostin plays an important role in the process of CSC niche formation [38, 53]. In addition, facilitate cancer stem cell (CSC) adhesion to the niche and protect CSCs from external differentiation stimuli, hence maintaining CSCs in undifferentiated state [53]. In lung metastases of the MMTV-PyMT mouse breast cancer model, CD90^+^ CSCs were observed to preferentially localize in the proximity of stromal niches and periostin-deficient animals exhibited a reduction in the CSC population [38]. In the MLL-AF9 acute myeloid leukemia (AML) in vivo model, it has been shown that CSCs express periostin and its receptor, integrin, in secondary target tissues indicating the autocrine and paracrine effects of periostin on CSCs behavior such as invasion and metastasis [147]. In addition they showed that periostin-deficient CSCs did not form tumor spheres and this effects could be reversed by addition of periostin. Consequently, it is implicated that periostin plays an important function in CSC maintenance and also metastatic niche modulation [147].

**Fig. 4:**
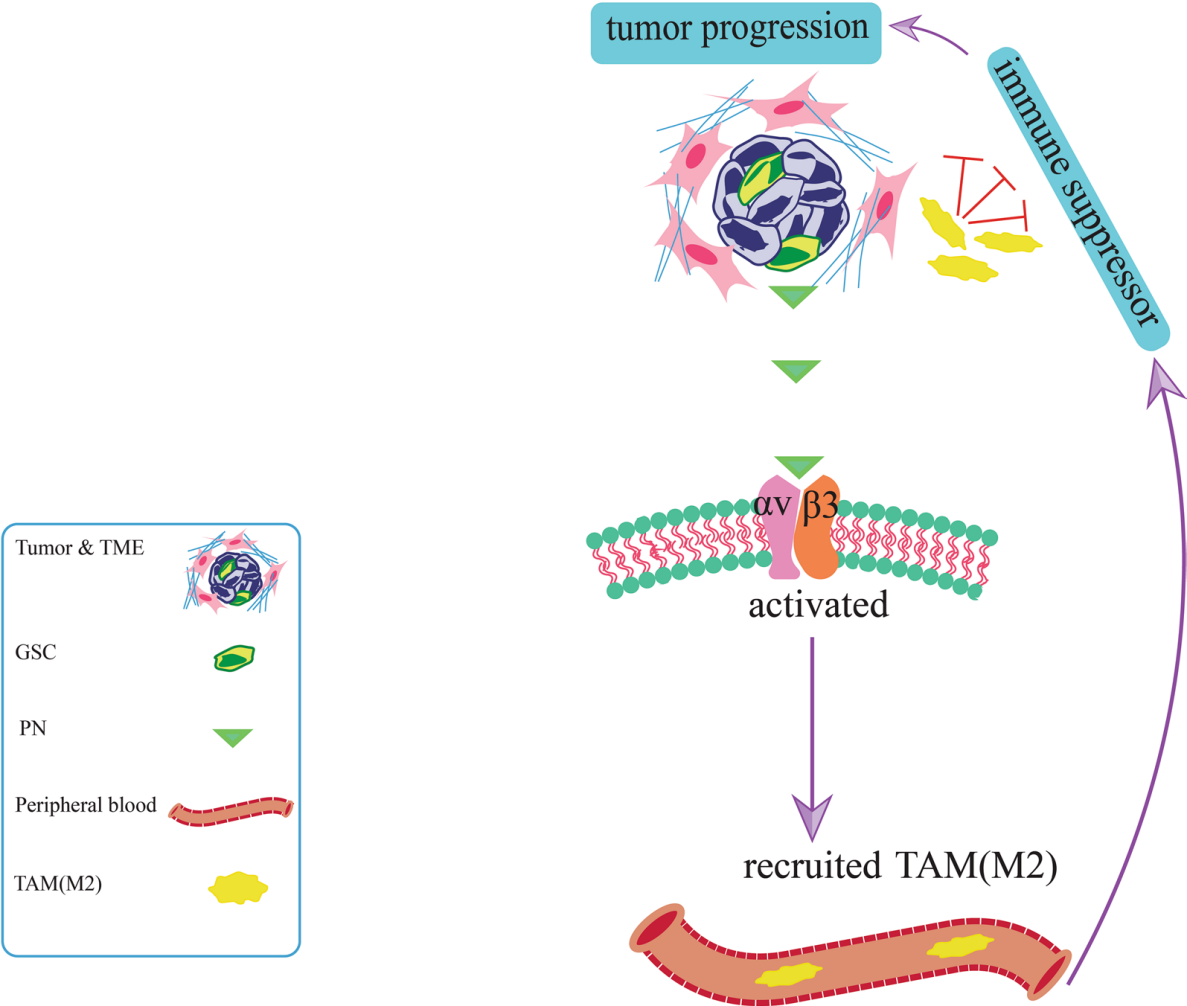
GSCs secrete periostin and activate the αvβ3 integrin signaling pathway, which recruits M2 tumor-associated macrophages from the peripheral blood to the tumor microenvironment. Tumor progression is aided by TAMs (M2)

In glioblastomas, perostin-secreted glioma stem cells (GSCs) recruits M2 tumor-associated macrophages (TAMs) from the peripheral blood to the TME through activation of the αvβ3 integrin signaling pathway and the immunosuppressive and tumor-supportive M2 tumor-associated macrophages are involved in tumor progression (Fig. 4) [148]. Elevated periostin expression is detected in basal-like breast cancer (BLBC), an aggressive subtype of breast cancer that consists mainly of CSCs [59]. Periostin interacts with Wnt1 and Wnt3A, resulting in a Wnt signaling pathway that promotes CSC-supporting niche formation and CSC maintenance, hence promoting metastasis [38, 131]. In addition to the Wnt signaling pathway, periostin can influence CSCs maintenance through activation of the periostin-integrin 3 signaling axis. Periostin activates the downstream of NF-κB transcription factor via the ERK signaling pathway or directly activates the NF-κB, consequently increasing IL-6 and IL-8 transcription and enhancing CSC maintenance (Fig. 3) [59].

## Periostin as a biomarker

Several studies have found that periostin overexpression in tumor stroma and cancer epithelial cells is associated with a more aggressive tumor, advanced stage or poor prognosis, and shorter overall survival in various cancers [11]. Now, there is a degree of incompatibility between periostin expression and clinico-pathological parameters in some types of cancer [51, 58]. In various studies on primary tumor/tumor cells/tumor stroma, no significant correlation was found between periostin expression and tumor size, age, or gender [54, 57, 60, 61, 65, 67, 70, 79]. In invasive ductal breast carcinoma (IDC), higher periostin expression levels in CAFs are associated with the grade of tumor cells and shorter overall survival, suggesting that periostin secreted by fibroblasts could be a marker in breast cancer progression [60]. Periostin was found to be upregulated in HNC, especially in patients with lymph node metastases. Periostin was overexpressed in the tissues of 79.3% of patients with HNC, as shown by RT-PCR. The overexpression of periostin was further confirmed by Western blotting. Consequently, periostin is an excellent biomarker for the prediction of metastasis in HNC patients [61]. Using RT–PCR and IHC analysis, It has been shown that Higher periostin expression is correlated with tumor grade, recurrence, progression, and shorter survival in human glioma patients [63] and periostin overexpression promotes OSCC invasion and angiogenesis. Periostin-positive tumor tissues have a higher blood vessel density than periostin-negative tumor tissues. Recombinant periostin also inccrease capillary formation in a concentration-dependent manner in OSCC cells, as described in an in vitro investigation. Therefore, periostin could be utilized to predict metastases in OSCC patients [7]. Up-regulation of periostin was validated in NPC stroma compared to normal nasopharyngeal (NNM) stroma by Western blotting and IHC, and significantly associated with clinical stages including lymph node metastases, and poor overall survival of nasopharyngeal cancer [67]. Furthermore, it was found that tumor stroma periostin- overexpressed NSCLC patients had a substantially worse survival rate than those with low-expression [116]. High periostin expression in tumor stroma is associated with decreased patient survival in pancreatic cancer [54] and prostate cancer [77, 149]. In summary, many studies in breast cancer and other epithelial cancers, such as NSCLC [116], pancreatic cancer [54], colon cancer [69], and prostate cancer [149], have found that a high expression of periostin in tumor stroma, particularly in CAFs, can be used as a prognostic biomarker [60].

Also, elevated periostin levels were detected in the serum of some cancer patients, suggesting that it could be a useful diagnostic and prognostic biomarker [138]. Nuzzo et al. reported a correlation between elevated serum periostin levels and breast cancer-specific mortality in a subgroup of patients who did not receive adjuvant systemic therapy. In addition, they suggested that serum periostin could be detected in early breast cancer patients prior to surgery, and higher serum base-line levels predicted worse long-term survival for specific patient subgroups [150]. In another study, there was no association between serum periostin levels and clinical outcome in breast cancer patients with or without bone metastases [50]. Serum periostin levels in 296 NSCLC patients were significantly higher than in healthy controls or patients with benign lung disease. Higher serum periostin levels were associated with poorer PFS and OS, indicating serum periostin levels as an independent prognostic marker [151]. In another study, serum periostin levels were significantly higher in NSCLC patients compared to patients with benign lung disease and healthy controls and serum periostin levels were correlated with bone metastases and chemotherapy response in patients. Thus, serum periostin levels may be used to predict chemotherapy efficacy and survival in NSCLC [152]. In another study, increased serum periostin levels were associated with poor prognosis, metastasis, and advanced disease (stage III/IV) in CRC patients. It was suggested that serum periostin levels may be useful in diagnosing CRC patients with high risk for metastasis [57]. The relationship between periostin expression and clinicopathological parameters and survival is presented in Table 5.

**Table 5:** The relationship between periostin expression and clinico-pathological parameters and survival

Cancer type	Tissue/Serum	Detection system						Periostin expression (%)	Main findings	Ref
		Real- time PCR	NB	WB	IHC	ELISA	MA			
Breast	Tissue (IDC)	+			+			70% of tumor cases	High level of PN expression in CAFs was correlated with grade and shorter overall survival	[60]
	Tissue	+		+	+				High level of epithelial PN expression was associated with reduced disease-free survival and overall survival	[48]
	Serum					CLIA method			High level of serum PN were observed in patients with bone metastases No correlation was found between serum PN levels and other prognostic factors including clinical stage or lymph node metastasis	[50]
	Serum					+			High level of serum PN had a significant correlation with patient age, adjuvant systemic therapy and PgR status	[150]
	Tissue				+				High level of tumor cell PN expression was significantly associated with HER2 status, histological grade, patient age, and lympho vascular invasion High level of tumor cell PN expression was significantly associated with reduced OS	[153]
	Serum					+			High level of serum PN had a significant correlation with patient OS and breast cancer-specific mortality PN expression is an independent prognostic marker for breast cancer-specific survival	[154]
Head and neck	Tissue	+		+	+	+		23 /29 (79.3%) of tumor cases	High level of PN expression associated with lymph node metastasis	[61]
Osteosarcoma	Tissue				+			55/68 (80.9%) of tumor cases	High level of PN expression associated with histological subtype, Enneking stage and tumor size, VEGF expression and higher microvessal density Patients with PN-positive expression had a significant shorter OS and DFS than patients with PN-negative expression PN expression, along with histological subtype, Enneking stage, and tumor size, is an independent prognostic factor for OS and DFS	[62]
Leiomyosarcoma	Tissue	+			+				High stromal expression of PN were associated with reduced OS	[155]
Glioma	Tissue	+		+	+				High level of PN expression in all grades of adult human glioma has a direct correlation with tumor grade and recurrence, and inversely correlates with survival PN mRNA expression was significantly higher in grade IV gliomas than in grade II and grade III tumors	[63]
Ovary	Tissue				+		+	(30.2%) high (69.8%)low	Patients with PN overexpression had a significant shorter OS and DFS than patients with low PN expression in stroma, as well as was associated with poor prognosis, platinum resistance, higher ratio of advanced FIGO stage, higher tumor recurrent incidence after first treatment	[65]
	Tissue		+		+		+		High level of PN expression had a significant correlation with clinical late stages (III/IV) and cancer recurrence	[66]
Oral squamous cell carcinoma	Tissue	+			+			21/31 (68%) RT–PCR 51/74 (69%) IHC	High level of PN expression significantly correlated with invasion pattern metastasis and angiogenesis (The density of blood vessels in PN -positive individuals was higher than in PN-negative cases.)	[7]
Cutaneous squamous cell carcinomas	Tissue				+				High level of PN stroma expression significantly correlates with poor prognosis	[68]
Nasopharyngeal carcinoma (NPC)	Tissue			+	+				High level of PN expression significantly correlated with tumor stage, histologic type/grade, recurrence, lymph node metastasis, and reduced OS High level of PN expression was an independent prognostic factor	[67]
Colorectal	Tissue		+		+			More than 80%	High level of PN expression associated with the metastasized	[69]
	Serum	+			+	+		12/15 (80%) IHC of tumor cases	High level of preoperative serum PN was correlated with distant metastasis, advanced-stage disease (stage III/IV) and poor prognosis Serum PN levels in CRC patients were considerably higher than in healthy volunteers and benign colorectal polyps or adenomas 15/67 cases had significantly higher preoperative serum PN levels than matched postoperative levels	[57]
	Tissue				+			72.97% (821/1125) Grade 0, 33/1125 (2.9%), Grade 1, 296/1125 (26.3%), Grade 2, 492/1125 (43.7%), Grade 3, 304/1125 (27.0%)	High level of PN expression in tumor stroma significantly associated with tumor location in proximal colon, undifferentiated histology, infiltrative growth pattern, tumor budding, luminal necrosis, and higher TNM stage High level of PN expression in tumor stroma was an independent prognostic factor for poor 5-year cancer-specific survival and 5-year PFS	[70]
	Tissue				+			218/720 (30.28%)	High level of PN expression had a linear correlation with tumor size, histological type, lymph node metastasis, TNM stage, and postoperative liver metastasis High PN expression was associated with CRC-specific survival in 682 cases PN, histological grade, lymph node metastasis and TNM stage were as independent prognostic factors	[47]
Non-small cell lung carcinoma	Serum					+			The level of serum PN had no correlation with gender, age, pathological type, TNM stage, lymph node status, tumor size and invasiveness The level of serum PN in patients had fallen significantly 4 weeks after the tumor was removed, almost 40% lower	[116]
	Serum					+			High level of serum PN significantly related to bone metastasis The median PFS for the 122 non–small cell lung cancer patients was 5 months	[152]
	Serum					+			Patients with higher level of serum PN had a poorer PFS and OS than lower PN group High level of serum PN was an independent prognostic factor High level of serum PN was significantly associated with stage, lymph node metastases, and distant metastases	[151]
	Tissue				+				High level of PN expression was associated with male gender, higher stage, higher pT category, and greater tumor size in both stroma and tumor epithelia, but only in stroma with tumor relapse High level of stromal PN expression inversely correlated with PFS and it was a prognostic factor for decreased PFS	[71]
	Tissue	+		+	+				The expression level of PN protein was higher in the cancer tissue than in normal and paratumor tissues but not at the mRNA level High level of PN expression significantly had an inverse association with survival time and a linear association with poor prognosis Expression level in the male patients was significantly higher compared with female patients at the protein and mRNA	[72]
	Tissue				+				High level of PN expression had an inverse association with OS PN was not an independent prognostic factor	[73]
	Tissue	+			+				High level of PN expression in CAF had a direct association with clinical cancer stage, grades (G), and lymph node involvement Higher levels of PN expression in CAFs were found to be an independent prognostic factor of OS	[156]
Pancreas	Tissue	+		+	+			up to 80% (strong positive in the neoplastic stroma) 30% (strong positive in neoplastic epithelium)	High level of PN expression in the neoplastic stroma significantly had a correlation with depth of invasion and lymph node metastasis PN expression in the stroma or epithelium was associated with poor survival	[54]
	Tissue	+		+	+				High level of PN expression had an association with shorter OS PN mRNA levels were 42-fold higher in cancer than in normal tissue, and had a strong correlation with protein levels	[8]
Prostate	Tissue			+	+				High level of PN expression positively correlated with Gleason score and the aggressiveness of prostate disease	[74]
	Tissue				+			142/418 (34.0%): Epithelial of carcinoma cells 11/ 38 (28.9%): Benign glands	High level of PN epithelial expression had a significant association with high Gleason score and advanced tumor stage High level of stroma PN expression had a significant association with higher Gleason score	[75]
	Tissue				+				High level of PN stroma expression significantly correlated with the malignancy degree (Gleason score) PN epithelial expression was increased in the early stages of PCa (Gleason score 6–7) but not in the progressive stages of PCa	[76]
	Tissue				+				High level of PN stroma expression significantly correlated with reduced survival, while low level of PN epithelial expression significantly correlated with reduced PSA-free survival PN stromal expression had a poor correlation with Gleason score PN epithelial expression significantly correlated with extra-prostatic extension	[77]
					+				High level of PN stroma expression significantly correlated with poor prognosis and the lack of statistical significance between epithelial PN expression and OS	[157]
Bladder	Tissue	+	+						Downregulation of PN mRNA was significantly correlated with higher grade and stage PN mRNA expression was shown to be more common in bladder cancers with a low stage pTa (12/18; 66.6 percent) than those with a high stage pT1–3 (5/15; 33.3 percent)	[58]
	Tissue			+	+				PN mRNA levels were significantly elevated in patient MIBC tissue vs NMIBC and normal tissue - Means MIBC PN level was 70 times higher than healthy controls Epithelial PN expression in MIBC tissues was associated with reduced progression-free and disease-specific survival (poor clinical outcome) Patients whose MIBC cells had PN had a significantly higher risk for tumor development and disease-specific mortality Mild to high (2+/3+) level of PN expression significantly correlated with disease progression in patients with NMIBC	[56]
Liver	Tissue				+			HCC: 19/91 (20.9%—strong epithelial) and 10/91 cases (11%—strong stroma) BDC: 39/116 (33.6%—strong epithelial) and 78/116 (67.2%—strong stroma)	High level of PN epithelial expression had a significant association with higher tumor grade, reduced OS, younger age at diagnosis, female gender and HBV infection PN expression were independent of pT level, differentiation grade, or proliferation rate in primary BDC	[78]
	Tissue				+				PN levels were higher in patients with multiple tumors, positive microvascular invasion, and advanced stage disease High PN expression level had a significant inverse association with overall survival	[79]

IDC: Invasive ductal breast carcinoma; CLIA: Chemiluminescence immunoassay; PgR: Progesterone receptor; OS: Overall survival; DFS: Disease-free survival; PFS: Progression-free survival; BLD: Bbenign lung diseases; PCa: Prostate cancer; MIBC: Muscle-invasive bladder cancer; EVs: Extracellular vesicles; NMIBC: Non-muscle-invasive bladder cancer; HCC: Hepatocellular carcinoma; BDC: Bile duct carcinomas; WB: Western blotting; IHC: Immunohistochemistry; ELISA: enzyme-linked immunosorbent assay; MA: Microarray analysis; NB: Northern blot; IB: Immunoblot; CLIA: Chemiluminescence immunoassay

## Periostin as a therapeutic target in cancer

Given the well-known role of periostin in cancer development, Periostin targeted therapy could be an effective treatment approach. Several studies have introduced effective methods for blocking periostin signaling pathways in preclinical models of cancer. In glioblastoma, for instance, there is a negative association between periostin expression and miR-599, and overexpression of miR-599 inhibits glioma cell motility and invasion by down-regulating periostin expression. Consequently, miR-599 could be utilized to suppress periostin expression in human gliomas [158].

Some studies have shown that blocking of periostin function by anti-periostin antibodies are an effective cancer treatment strategy. Anti-periostin neutralizing antibody (PN1-Ab) has been developed against peptide encoded by exon 17. In a mouse model of breast cancer, PN1-Ab inhibited the growth of primary tumors and metastatic lesions as well as bone degradation, resulting an increase in the survival rate [6]. Zhu et al. found that in ovarian mice model, anti-periostin monoclonal antibodies (MZ-1) decreased the number of metastatic ovarian lesions [159]. In addition, in breast cancer mouse model, tumor growth and metastasis were inhibited using benzyl-d(U)TP-modified DNA aptamers (PNDAs) targeted human periostin [160]. It was also recently revealed that a small segment of periostin including exon 17 but not exon 12 tightly binds wnt3a, and the blocking antibody may inhibit the development of primary and metastatic breast cancer [114]. MPC5B4 monoclonal antibody, which inhibits the interaction between periostin and avb3 integrin, was also engineered to detect amino acids 136–51 within the periostin fascilin (FAS) 1–1 domain to inhibit periostin function. Breast tumor cell periostin expression was reported to be a powerful prognostic indicator and to correlate with tumor size, lymph node status, and human epidermal growth factor receptor 2 (HER2) status [153]. Consequently, these therapeutic approaches can be used for cancer treatment.

## Conclusion

Beyond the physiological role of periostin, the cumulative information about its function in cancer over the last decades suggests that this matricellular protein can be a key player in cancer growth and progression, emphasizing the importance of our efforts to better understand the structure and function of this molecule. Some lines of evidence indicate that periostin has a fundamental role in cancer cell proliferation and survival, EMT, per-metastastatic nichs formation, CSC niche establishment, cell migration and ECM remodeling, modulation of immune cells in PMN, and chemoresistance, the most important hallmarks of cancer.

As mentioned above, this molecule plays a key role in various processes of tumorigenesis and metastasis. From a therapeutic point of view, chemoresistance is the main obstacle to cancer treatment. Several mechanisms have been implicated in drug resistance. Of note, periostin promotes chemoresistance through several mechanisms, including: 1-induction of EMT; 2-increased cancer cell stemness; 3-upregulation of LOX and increasing stiffness; 4-activation of Akt and Erk, the PI3K/Akt/survivin, and the PI3K/Akt/survivin pathways. In addition, overexpression of periostin was investigated in a variety of malignancies and indicated a relationship between the increased expression of periostin and clinico-pathological characteristics. The function of this molecule in tumor progression along with its expression in cancerous tissues and its low expression in normal tissues makes periostin an attractive therapeutic target.

However, several questions related to the function of various isoforms of periostin, especially in cancer, tissue distribution of isoforms, overexpression in cancer tissues, and interaction with different molecules such as integrins, remain unanswered. Addressing these questions will be crucial to understanding how periostin functions and to developing new treatment strategies for cancer.

## Data Availability

Not applicable.

## Abbreviations

ECM: Extracellular matrix
EMT: Epithelial-mesenchymal transition
CDNA: Complementary DNA
ORF: Open reading frame
NSCLC: Non-small cell lung cancer
PLF: Periostin-like-factor
FAK: Focal adhesion kinase
FAS1: Fasciclin-like
GPI: Glycosyl phosphatidyl inositol
MFAS: Midline fasciclin
CTD: Carboxyl-terminal domain
CCN3: Cellular Communication Network Factor 3
TGFBIp: TGF-β-induced protein
BMP-1: Bone morphogenetic protei-1
PBLs: Peripheral blood lymphocytes
PVR: Proliferative vitreoretinopathy
IPF: Idiopathic pulmonary fibrosis
CRC: Colorectal cancer
DCIS: Ductal carcinoma in situ
IBC: Invasive breast cancer
P73: Tumor protein 73
CTR: C-terminal region
ER: Endoplasmic reticulum
NPC: Nasopharyngeal carcinoma
IBC: Invasive breast carcinoma
CAF: Cancer-associated fibroblast
NFs: Normal fibroblasts
LOX: Lysyl oxidase
RCC: Renal cell carcinoma
HSC2: Human Oral Squamous Carcinoma cell line2
HSC3: Human Oral Squamous Carcinoma cell line3
VEGF-C: Vascular endothelial growth factor-C
EGFR: Epidermal growth factor receptor
MMP-9: Matrix metalloproteinase-9
ILK: Integrin-integrin-linked kinase
NF-κB: Nuclear factor κB
CCL2: Chemokine ligand 2
CSCs: Cancer stem cells
AML: Acute myeloid leukemia
GSCs: Glioma stem cells
TAM: Tumor-associated macrophages
BLBC: Basal-like breast cancer
IDC: Invasive ductal breast carcinoma
HNC: Head and neck cancer
OSCC: Oral squamous cell carcinoma
NPC: Nasopharyngeal carcinoma
NNM: Normal nasopharyngeal
ELISA: Enzyme-linked immunosorbent assays
EGFR: Epidermal growth factor receptor
ILK: Integrin-linked kinase
